# The skeletal muscles of mice infected with *Plasmodium berghei* and *Plasmodium chabaudi* reveal a crosstalk between lipid mediators and gene expression

**DOI:** 10.1186/s12936-020-03332-3

**Published:** 2020-07-14

**Authors:** Mauro Toledo Marrelli, Zhiying Wang, Jian Huang, Marco Brotto

**Affiliations:** 1 Department of Epidemiology, School of Public Health, University of São Paulo, Avenida Dr. Arnaldo 715, São Paulo, SP 01246-904 Brazil; 2 Bone-Muscle Research Center, College of Nursing and Health Innovation, University of Texas-Arlington, 655 W. Mitchell Street, Arlington, TX 76010 USA

**Keywords:** Malaria, Skeletal muscles, Muscle damage, Lipid signaling mediators, Gene array

## Abstract

**Background:**

Malaria is one of the most prevalent infectious disease in the world with 3.2 billion humans at risk. Malaria causes splenomegaly and damage in other organs including skeletal muscles. Skeletal muscles comprise nearly 50% of the human body and are largely responsible for the regulation and modulation of overall metabolism. It is essential to understand how malaria damages muscles in order to develop effective preventive measures and/or treatments. Using a pre-clinical animal model, the potential molecular mechanisms of *Plasmodium* infection affecting skeletal muscles of mice were investigated.

**Methods:**

Mouse Signal Transduction Pathway Finder PCR Array was used to monitor gene expression changes of 10 essential signalling pathways in skeletal muscles from mice infected with *Plasmodium berghei* and *Plasmodium chabaudi*. Then, a new targeted-lipidomic approach using liquid chromatography with tandem mass spectrometry (LC–MS/MS) to profile 158 lipid signalling mediators (LMs), mostly eicosanoids derived from arachidonic acid (AA), eicosapentaenoic acid (EPA) and docosahexaenoic acid (DHA), was applied. Finally, 16 key LMs directly associated with inflammation, oxidative stress, and tissue healing in skeletal muscles, were quantified.

**Results:**

The results showed that the expression of key genes altered by *Plasmodium* infection is associated with inflammation, oxidative stress, and atrophy. In support to gene profiling results, lipidomics revealed higher concentrations of LMs in skeletal muscles directly related to inflammatory responses, while on the levels of LMs crucial in resolving inflammation and tissue repair reduced significantly.

**Conclusion:**

The results provide new insights into the molecular mechanisms of malaria-induced muscle damage and revealed a potential mechanism modulating inflammation in malarial muscles. These pre-clinical studies should help with future clinical studies in humans aimed at monitoring of disease progression and development of specific interventions for the prevention and mitigation of long-term chronic effects on skeletal muscle function.

## Background

Malaria remains as the most significant human infectious diseases in the World with 3.2 billion humans at risk [1]. The disease continues to spread and curb the development of various African countries, causing a huge economic and social cost [2]. Malaria pathogenesis is a process by which malaria parasites cause illness, abnormal function and damage in the hosts. The malaria pathogens have been related to cause detrimental effects on skeletal muscles of animals and humans [3–9]. In addition of affecting skeletal muscles, severe malaria may cause damage on cardiac muscles [10–12]. Circulation levels of cardiac proteins increases with the severity of malaria, indicating myocardial impairment in complicated falciparum malaria [13, 14].

The skeletal muscle microvascular function and its oxygen consumption is proportionally impaired to the disease degree. Strikingly, oxygen consumption in severe malaria reduces similarly as in sepsis patients [15]. RNA and protein contents in much lower levels can be found in malaria infected skeletal muscles compared with non-infected controls [16]. Previous study demonstrated that in mice infected with *Plasmodium berghei*, both glycolytic and oxidative hind limb skeletal muscles produced half of the normal contractile force, fatigued significantly more, and recovered significantly less [7]. These effects were associated with a reduced content of key contractile proteins, such as troponins and myosin [7]. Although these results could help to explain many of the symptoms in humans infected with malaria, they do not explain the molecular and cellular pathway mechanism of direct damage to the contractile machinery. The investigation of genes expression changes and of other biomolecules involved on the physiological effects of the malaria pathogen on muscles becomes crucial to understand potential mechanisms underlying this musculoskeletal disease. Involved in many physiological processes, the lipid signalling mediators, a class of bioactive metabolites of the essential polyunsaturated fatty acids (PUFA) [17–22], have different major functions in living systems, such as being major constituents of biological membranes, efficient energy sources, modifiers of proteins, and signalling molecules [18]. These signalling molecules generate locally through specific biosynthetic enzymes/receptors in response to extracellular stimuli, and play an important role through their signalling pathways on the regulation of pathophysiological states such as inflammation, metabolic syndrome, and cancer [18, 23, 24]. Thus, some LMs could serve as biomarkers in therapy or diagnosis of diseases, as well as potential candidates in drug development.

Herein, to study the molecular mechanisms of malaria-induced injury, 10 gene-signalling pathways were simultaneously monitored in mouse skeletal muscles infected with *Plasmodium berghei* and *Plasmodium chabaudi*. PCR Arrays are reliable tools for analysing focused panels of genes in signal transduction, biological processes or disease research pathways. Furthermore, a new targeted lipidomic approach using LC–MS/MS method, developed by the authors, was applied to profile total 158 LMs, and further quantify 16 key LMs directly associated with inflammation and tissue healing in skeletal muscles. The results showed a high level of concordance between the RT-PCR gene arrays and lipidomics. In the course of malaria, muscles present a conclusive signature of enhanced inflammation signalling and reduced signalling to resolve inflammation. More specifically, a crosstalk between arachdonic acid (AA) related lipid mediators and *Acsl*4 gene was revealed as a potential mechanism modulating inflammation in muscles during *Plasmodium* infection.

## Methods

### Chemicals and reagents

Sixteen isotope-labelled lipid mediator (LM) internal standards (IS) including arachidonic acid-d_8_ (AA-d_8_), 6-keto prostaglandin F_1α_-d_4_ (6-keto-PGF_1α_-d_4_), prostaglandin F_2α_-d_4_ (PGF_2α_-d_4_), prostaglandin E_2_-d_4_ (PGE_2_-d_4_), prostaglandin D_2_-d_4_ (PGD_2_-d_4_), thromboxane B_2_-d_4_ (TXB_2_-d_4_), leukotriene B_4_-d_4_ (LTB_4_-d_4_), leukotriene C_4_-d_5_ (LTC_4_-d_5_), 5S-hydroxy-6E,8Z,11Z,14Z-eicosatetraenoic-5,6,8,9,11,12,14,15-d_8_ acid (5-HETE-d_8_), 15S-hydroxy-5Z,8Z,11Z,13E-eicosatetraenoic-5,6,8,9,11,12,14,15-d_8_ acid (15-HETE-d_8_), and 12S-hydroxy-5Z,8Z,10E,14Z-eicosatetraenoic-5,6,8,9,11,12,14,15-d_8_ acid (12-HETE-d_8_), platelet-activating factor C-16-d_4_ (PAF C-16-d_4_), tetranor-prostaglandin E metabolite-d_6_ (tetranor-PGEM-d_6_), oleoyl ethanolamide-d_4_ (OEA-d_4_), docosahexaenoic acid-d_5_ (DHA-d_5_), and eicosapentaenoic acid- d_5_ (EPA-d_5_), and eighteen LM standard compounds including 6-keto-PGF_1α_, PGF_2α_, PGE_2_, PGD_2_, PGA_2_, TXB_2_, LTB_4_, LTC_4_, 12(S)-hydroxyheptadecatrienoic acid (12-HHT), 9-hydroxy-10,12-octadecadienoic acid (9-HODE), 13(S)-hydroxyoctadecadienoic acid (13-HODE), 17,18-dihydroxy-5Z,8Z,11Z,14Z-eicosatetraenoic acid (17,18-DiHETE), 8-hydroxy-4Z,6E,10Z,13Z,16Z,19Z-docosahexaenoic acid (8-HDoHE), N-arachidonoylethanolamine (AEA), EPA, DHA, OEA and AA were purchased from Cayman Chemical Co. (Ann Arbor, MI). Formic acid (reagent grade, ≥ 95%) was obtained from Sigma–Aldrich (St. Louis, MO, USA). HPLC–MS grade acetonitrile, water, methanol, and ethanol were purchased from J.T. Baker (Phillipsburg, NJ, USA). Tri‐reagent from Molecular Research Center, Inc. (Cincinnati, OH, USA). High‐capacity cDNA reverse‐transcription kit from Applied Biosystems (Foster City, CA, USA). Mouse Signal Transduction Pathway Finder PCR Array. RT2 First Strand Kit. RT2 Real‐Time SYBR green/Rox PCR master mix from SABiosciences (Valencia, CA, USA). RNeasy Mini Kit from Qiagen (Valencia, CA, USA).

### Animals and infection with malaria

Male mice from 4 to 5 months of age (Swiss Webster), weighing between 25 and 30 g were infected with malaria parasites *P. berghei* (strain ANKA 2.34) and *P. chabaudi* (strain CR), uninfected mice used as controls of the experiments. *Plasmodium berghei* (strain ANKA) isolated from rats *Grammomys surdaster* 2.34, has been maintained by Swiss Webster mice passage in the laboratory, and *P. chabaudi* in Balb/c mice, as described by Hoffmann et al. [25]. For the infections, the mice were intravenously inoculated with identical rates (200 µl) frozen red cell with 25% parasitaemia of malaria parasites diluted in PBS. The parasitaemia was monitored daily by microscopic examination of blood smears stained with Giemsa. Mice with parasitaemia of approximately 35% were selected for the analyses, based on previous findings [7], that these levels of parasitaemia lead to muscle dysfunction, but with no signs of brain dysfunction (i.e. deviation of head, seizures and coma followed by death). Therefore, in this study, only the mice without symptoms of cerebral malaria were used. These levels of parasitaemia were achieved 3-6 days after injection of parasites. Mice were euthanized by cervical dislocation and then intact muscles (gastrocnemius) were isolated, kept stabilized in RNA later and shipped to the Bone-Muscle Research Center, UT-Arlington. (https://www.uta.edu/conhi/research/bmrc/index.php).

### RNA isolation and RT‐PCR gene arrays

The Mouse Signal Transduction Pathway-Finder PCR Array from SABiosciences was used to simultaneously detect gene expression changes of 10 signalling pathways (see Table 1, and details in Results). Total RNA was extracted from the cells using the Tri reagent according to manufacturer’s protocol, quantified in a Nanodrop spectrophotometer (Thermo Scientific, Wilmington, DE, USA) by determining absorbance at 260 nm in triplicate. RNA purity was indicated by the A260/280 nm absorbance ratio of 1.9 to 2.1 and A260/230 nm absorbance ratio ≥ 1.8. Using 0.5 μg of RNA, each sample was reverse transcribed in a 20‐µL reaction volume. cDNA was synthesized using the RT2 First Strand Kit and the PCR Array was run according to the manufacturer’s protocol, including a threshold of 0.25 and validation of each gene tested by the identification of single peaks in melting curves. As above, data were analysed using RT2 Profiler PCR Array Data Analysis Software (SABiosciences); CT values were normalized to built‐in six reference housekeeping genes, genomic DNA control, reverse transcription control, and positive PCR control [26]. This analytical software was used to set the statistical significance of upregulation or downregulation of all tested genes at fivefold difference. A major advantage of this technology is the pre-validation of these pathways at the protein level by the manufacturer and the robust utilization of internal and reference controls.

**Table 1:** Signalling pathways and genes in Mouse Signal Transduction Pathway Finder PCR Array

Signalling pathways	Genes
TGFß pathway	*Atf*4*, Cdkn*1b (*p27Kip*1)*, Emp*1*, Gadd*45b*, Herpud*1*, Ifrd*1*, Myc, Tnfsf*10
WNT pathway	*Axin*2*, Ccnd*1*, Ccnd*2*, Dab*2*, Fosl*1 (*Fra*-1)*, Mmp*7 (*Matrilysin*)*, Myc, Ppard, Wisp*1
NFκB pathway	*Bcl*2a1a (*Bfl*-1/A1)*, Bcl*2l1 (*Bcl*-x)*, Birc*3 (*c*-IAP1)*, Ccl*5 (*Rantes*)*, Csf*1 (*Mcsf*)*, Icam*1*, Ifng, Stat1, Tnf*
JAK/STAT pathway JAK1, 2/STAT1 STAT3 STAT5 JAK1, 3/STAT6	*Irf*1 *Bcl*2l1 (*Bcl*-x)*, Ccnd*1*, Cebpd, Lrg1, Mcl*1*, Socs*3 *Bcl*2l1 (*Bcl*-x)*, Ccnd*1*, Socs*3 *Fcer*2a*, Gata*3
p53 pathway	*Bax, Bbc*3*, Btg*2*, Cdkn*1a (*p*21*Cip*1*/Waf*1)*, Egfr, Fas* (*Tnfrsf*6)*, Gadd*45a*, Pcna, Rb*1
Notch pathway	*Hes*1*, Hes*5*, Hey*1*, Hey*2*, Hey*l*, Id*1*, Jag*1*, Lfng, Notch*1
Hedgehog pathway	*Bcl*2*, Bmp*2*, Bmp*4*, Ptch*1*, Wnt*1*, Wnt*2b*, Wnt*3a*, Wnt*5a*, Wnt*6
PPAR pathway	*Acsl*3*, Acsl*4*, Acsl*5*, Cpt*2*, Fabp*1*, Olr*1*, Slc*27a4*, Sorbs*1
Oxidative stress	*Fth*1*, Gclc, Gclm, Gsr, Hmox*1*, Nqo*1*, Sqstm*1*, Txn*1*, Txnrd*1
Hypoxia	*Adm, Arnt, Car*9*, Epo, Hmox*1*, Ldha, Serpine*1 (PAI-1), *Slc*2a1*, Vegfa*

### Lipidomics

All components of LC–MS/MS system are from Shimadzu Scientific Instruments, Inc. (Columbia, MD, USA). LC system was equipped with four pumps (Pump A/B: LC-30AD, Pump C/D: LC-20AD XR), a SIL-30AC autosampler (AS), and a CTO-30A column oven containing a 2-channel six-port switching valve. The LC separation was conducted on a C8 column (Ultra C8, 150 × 2.1 mm, 3 µm, RESTEK, Manchaca, TX, USA) along with a Halo guard column (Optimize Technologies, Oregon City, OR, USA). The MS/MS analysis was performed on Shimadzu LCMS-8050 triple quadrupole mass spectrometer. The instrument was operated and optimized under both positive and negative electrospray and multiple reaction monitoring modes (± ESI MRM). The settings of flow rate and gradient program for the LC system as well as MS/MS conditions are recommended by a software method package for 158 lipid mediators (Shimadzu Scientific Instruments, Inc., Columbia, MD, USA) and further optimized following previously published quantification method [27]. All analyses and data processing were completed on Shimadzu LabSolutions V5.91 software (Shimadzu Scientific Instruments, Inc., Columbia, MD, USA).

### Tissue preparation for lipidomics analyses

Striated muscles were isolated after mice sacrifice, then snap frozen in liquid nitrogen immediately and stored at -80 °C. Before the experiment, the aliquoted frozen muscle tissue (50–100 mg) were defrosted on ice and in the dark, weighed carefully, and minced into small pieces on ice. The minced muscle was placed into a 2.0 mL round-bottom low retention microcentrifuge tube (Fisher Scientific, Waltham, MA, USA) containing one 5 mm stainless steel bead (Qiagen, Germantown, MD, USA), and 1.0 mL of ice-cold 80% methanol in water (v/v) will be added. The mixture was homogenized using a Tissue Lyser II homogenizer (Qiagen, Germantown, MD, USA) at the frequency of 30 s^−1^, in 8 × 30-s bursts, waiting 20 s in between to avoid high temperature. The obtained homogenate was mixed with 5 µL of isotope-labelled LM internal standards (IS) mixture stock solution (5 µg/mL for AA-d_8_, 2 µg/mL for DHA-d_5_ and EPA-d_5_, and 0.5 µg/mL for the rest IS), and then agitated on ice and in the dark for 1–2 h, followed by centrifugation at 6000×*g* at 4 °C for 10 min to remove any tissue residue and precipitated proteins.

All muscle samples need to be cleaned and concentrated by Solid Phase Extraction (SPE) before being injected into LC–MS/MS. Ice-cold 0.1% formic acid (4 mL) was added in the obtained supernatant to fully protonate the LM species before sample was loaded to the preconditioned SPE cartridges (Strata-X 33 µm polymeric reversed phase, Phenomenex, Torrance, CA, USA). Once the sample had been totally loaded, cartridges were washed with 0.1% formic acid followed by 15% (v/v) ethanol in water to remove excess salts. Then the LMs from the SPE sorbent bed were eluted by methanol. Solvents were removed using an Eppendorf^®^ 5301 concentrator centrifugal evaporator (Eppendorf, Hauppauge, NY, USA). The dried extracts were stored at − 80 °C immediately for future LC–MS/MS analysis.

### Statistics analysis

For lipidomics analyses data is presented as mean ± SD of all samples in multiple experiments. One-way ANOVA with post hoc Tukey’s test (α = 0.05) was performed for data analysis. Differences were considered statistically significant at *p* < 0.05.

## Results

### RT-PCR Array

Table 1 shows the 10 signalling pathways and the specific genes monitored in each pathway through the Mouse Signal Transduction Pathway-Finder PCR Array from SABiosciences. This methodology was implemented to simultaneously detect gene expression changes in striated muscles of mice infected with *P. chabaudi* or *P. berghei*.

**Fig. 1:**
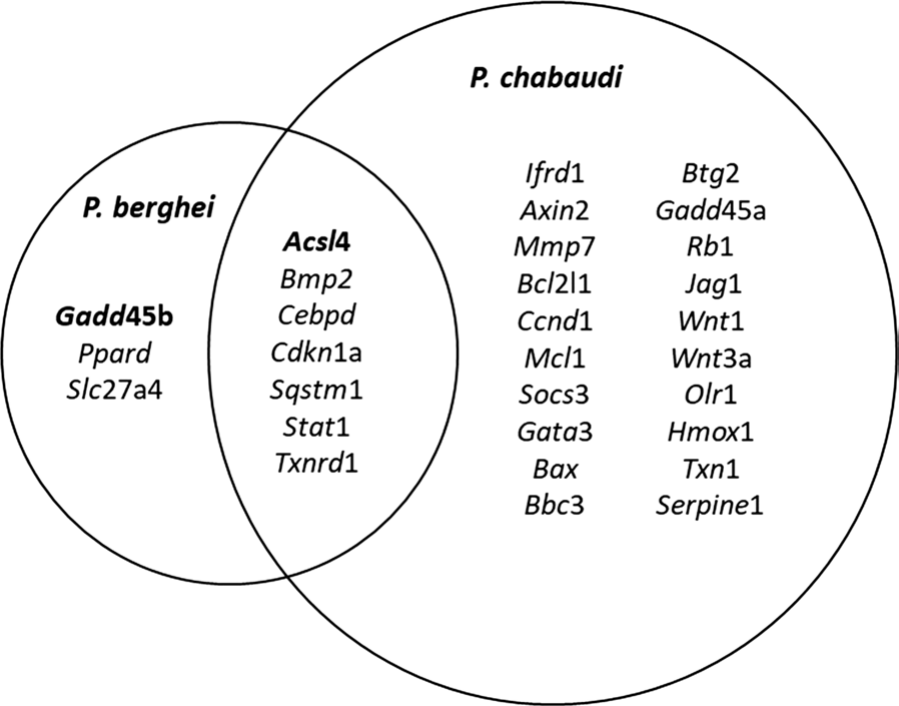
Venn diagram showing the common genes with altered expression in skeletal muscles from mice infected with either *P. chabaudi* or *P. berghei* parasites

The analysis showed three genes (*Gadd*45b*, Ppard, Slc*27a4) were significantly up-regulated compared to controls in skeletal muscles from mice infected with *P. berghei,* while twenty other genes (*Ifrd*1, *Axin*2*, Mmp*7*, Bcl*2l1*, Ccnd*1, *Mcl*1*, Socs*3, *Gata*3, *Bax, Bbc*3*, Btg*2, *Gadd*45a*, Rb*1, *Jag*1*, Wnt*1*, Wnt*3a, *Olr*1*, Hmox*1*, Txn*1, *Serpine*1) were up-regulated only in muscles from mice infected with *P. chabaudi* (Fig. 1).

**Fig. 2:**
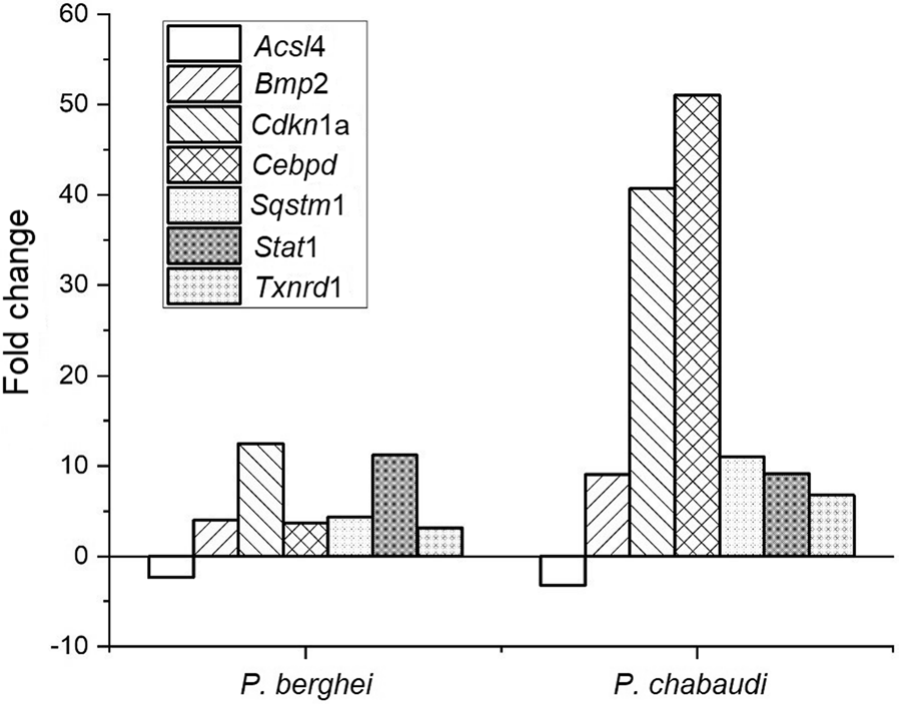
Key genes altered regulating inflammatory signalling pathways are present in skeletal muscles from mice infected with *P. chabaudi* and *P. berghei* parasites

Seven common genes (*Acsl*4, *Bmp2, Cebpd, Cdkn*1a, *Sqstm*1*, Stat*1 and *Txnrd*1) had their expression significantly altered in skeletal muscles from mice infected with either *P. chabaudi* or *P. berghei*, comparing to uninfected mouse controls, with higher expressions on *P. chabaudi* infected mice, as illustrated in Fig. 2.

Compared to negative control, the expression of *Acsl*4 gene was downregulated by -2.37‐fold (*p *= 0.005674) and -3.22‐fold (*p *= 0.018156), respectively, in muscles infected with *P. berghei* and *P. chabaudi*, respectively (Fig. 2). The other six genes were upregulated comparing to the uninfected mouse muscles, with *Bmp*2 gene altered by 3.99-fold (*p *= 0.012177) and 9.04-fold (*p *= 0.006948), respectively in *P. berghei* and *P. chabaudi* infected muscles. *Cebpd* was significantly altered by 3.72-fold (*p *= 0.025971) and 51.02-fold (*p *= 0.031599) in *P. berghei* and *P. chabaudi* infected muscles, respectively.

The expression of *Cdkn*1a gene was significantly up-regulated by 12.48-fold (*p *= 0.047676) and 40.70-fold (*p *= 0.009800), respectively in *P. berghei* and *P. chabaudi* infected muscles. *Stat*1 gene was significantly altered by 11.20‐fold (*p *= 0.021470) in *P. berghei* infected muscles, and 9.16-fold (*p *= 0.005999), in *P. chabaudi* infected muscles. Regarding the gene *Sqstm*1, it was upregulated related to the controls, by 4.33-fold (*p *= 0.008631) and 11-fold (*p *= 0.022897), respectively, in *P. berghei* and *P. chabaudi* infected muscles. Finally, the expression of *Txnrd*1 gene upregulated by 3.14-fold (*p *= 0.030464) in *P. berghei* infected muscles and 6.77-fold (*p *= 0.009119) in *P. chabaudi* infected muscles.

### Lipid Signalling Mediators (LMs)

**Fig. 3:**
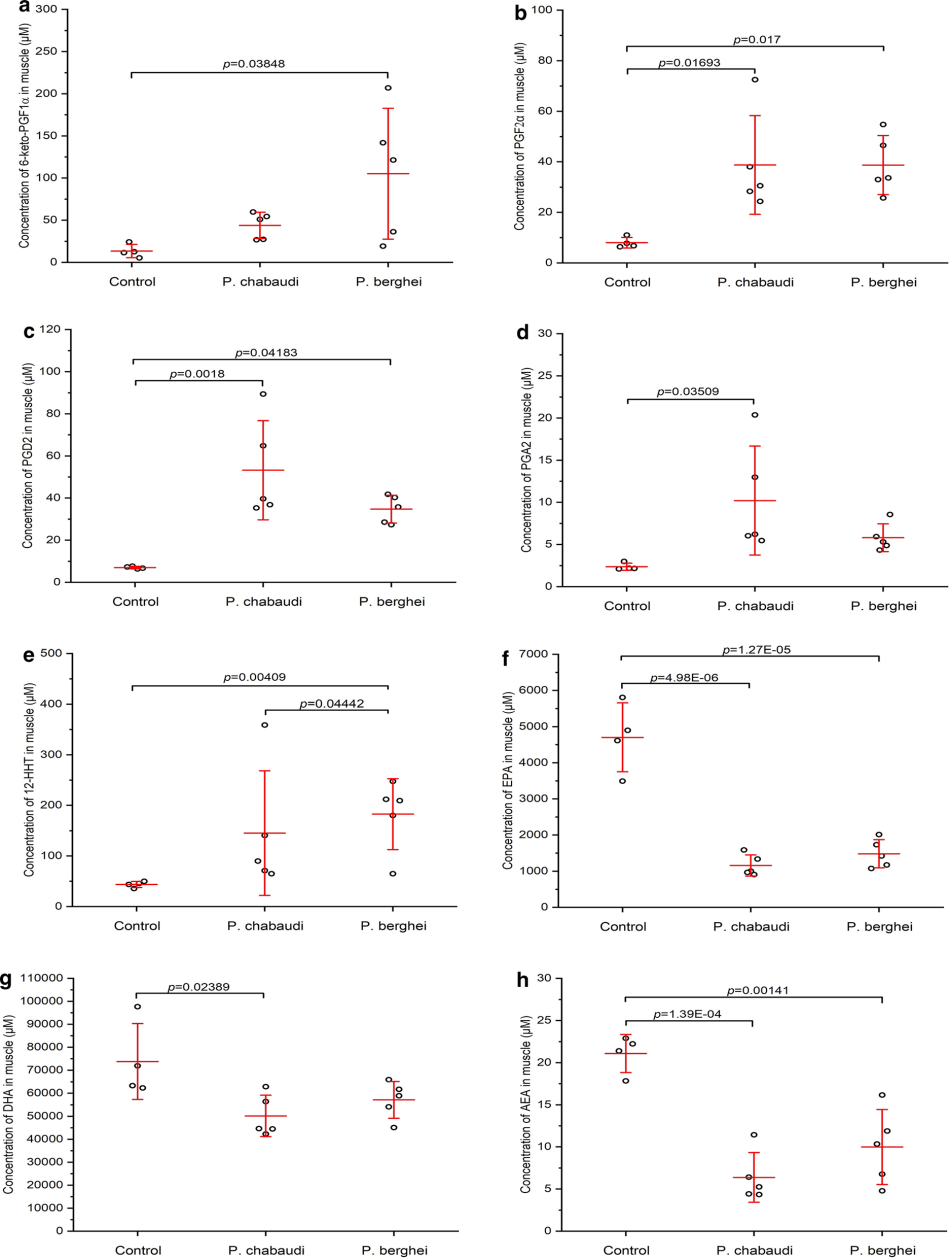
Comparison of the lipid concentration in muscles of control uninfected and infected mice with *P. chabaudi* and *P. berghei* parasites: **a** 6-keto-PGF_1α_, **b** PGF_2α_, **c** PGD_2_, **d** PGA_2_, **e**12-HHT, **f** EPA, **g** DHA, **h** AEA

Due to the essential roles of lipid signalling in health and disease, the profiles of total 58 lipid signalling mediators were compared between the muscles from mice infected with the two malaria parasites and uninfected mice (control) through a targeted LC–MS/MS based targeted lipidomics analytical method. Then the absolute concentrations of 16 key LMs, was further determined and related to six metabolic pathways, from mouse skeletal muscles in this study. This quantification method, developed in the Brotto Muscle Research Laboratory in collaboration with the Shimadzu Research Institute, was recently applied and showed substantial differences during aging as well sex-related differences in mice [27]. In the present study, both quantification and profiling results indicated significant differences (*p* < 0.05) in LM levels in skeletal muscles of mice infected with *P. berghei* and *P. chabaudi* compared to that of the muscles from the control mice (Table 2, Additional file 1: Table S1, and Fig. 3).

**Table 2:** Quantification of lipid mediator (pg mg ^−1^ muscle) in striated muscles from mice with or without different malaria infections and the associated metabolic pathways

Metabolic pathways	Lipid mediator	Control	*P. chabaudi*	*P. berghei*
Arachidonic acid (AA)	6-keto-PGF_1α_	*13.4 ± 7.9*	43.9 ± 15.6	105.1 ± 77.6*
	TXB_2_	3.4 ± 1.7	12.1 ± 12.8	26.9 ± 23.3
	PGF_2α_	*7.9 ± 2.1*	38.7 ± 19.5*	38.7 ± 11.7*
	PGD_2_	*7.0 ± 0.6*	53.2 ± 23.5*	34.7 ± 6.6*
	PGA_2_	*2.4 ± 0.4*	10.2 ± 6.4*	5.8 ± 1.6
	12-HHT	*43.7 ± 5.9*	145.1 ± 23.1*	182.7 ± 70**^, #^
	AA	16,644 ± 4727.6	21,033.5 ± 9395.7	22,340.5 ± 6285.2
Linoleic acid (LA)	13-HODE	911.6 ± 183.9	5724.6 ± 5486.6	1934.3 ± 989.8
	9-HODE	1159.2 ± 314.2	7427.6 ± 6820.3	2521.9 ± 1288.6
Eicosapentaenoic acid (EPA)	17,18-DiHETE	11.5 ± 1.4	8.6 ± 5.9	9.2 ± 4.1
	EPA	*4702.9 ± 954.4*	1160 ± 292.7***	1483.4 ± 391.1***
Docosahexaenoic acid (DHA)	8-HDoHE	136.8 ± 52.9	208.3 ± 85.6	221.9 ± 49.7
	DHA	*73,814.6 ± 16,486.1*	50,135.2 ± 9021.8*	57,149.6 ± 8011.9
α-Linolenic acid (ALA)	9-HOTrE	16.9 ± 1.2	121.4 ± 110.5	42.1 ± 13.8
Ethanolamide (EA)	AEA	*21.1 ± 2.2*	6.4 ± 2.9***	9.9 ± 4.4***
	OEA	36.5 ± 10.5	62.9 ± 22.2	72.1 ± 25
Ratio of AA/EPA		*3.6 ± 0.8*	19.9 ± 11.9*	16.7 ± 7.7

Mean ± SD, n = 5. One-way ANOVA with Tukey post hoc test (α = 0.05) was applied to compare means between multiple groups. *p < 0.05; **p < 0.01; ***p < 0.001 represent statistically significant difference between uninfected mice (Control) and *P. berghei* or *P. chabaudi* infected mice; ^#^*p *< 0.05 represents statistically significant difference between *P. berghei* and *P. chabaudi* infected mice

In 16 quantified LMs, the muscle levels of four LMs derived from arachidonic acid (AA, 20:4, ω-6), including 6-keto-PGF_1α_, PGF_2α_, PGD_2_, 12-HHT, were observed to be remarkably higher in mice infected with *P. berghei* than uninfected mice. As for the two LMs showing significantly lowered concentrations, EPA belongs to ω-3 PUFA eicosapentaenoic acid (EPA, 20:5, ω-3) metabolic pathway, and AEA belongs to fatty acid ethanolamide (EA)-derived metabolic pathway (Table 2, Fig. 3). Analysis of LM quantification from muscles of *P. chabaudi* infected mice showed similar results. The concentrations of 4 LMs from AA metabolic pathway, including PGF_2α_, PGD_2_, PGA_2_ and 12-HHT, were significantly higher than the controls, while two ω-3 PUFA metabolites from docosahexaenoic acid (DHA, 22:6, ω-3) and EPA pathways (EPA and DHA), and one EA metabolite (AEA) were found in lower concentrations comparing to the muscles from uninfected mice (Table 2, Fig. 3).

All LMs related to ω-6 PUFA metabolic pathways, including both AA and linoleic acid (LA, 18:2, ω-6) determined in this study, exhibited elevated levels in striated muscles from mice with two types of malaria, even though no statistically significant differences (*p *< 0.05) were observed (Table 2). Two hydroxyoctadecadienoic acids 13-HODE and 9-HODE, being considered as lipid peroxidation biomarkers [28, 29], showed much higher concentrations in malaria infected muscles, about 6-fold (*P. chabaudi*) and twofold (*P. berghei*) higher than the control, respectively, for both 13-HODE and 9-HODE (Table 2).

The AA/EPA ratio, an indicator of inflammation and skeletal muscle depletion [30, 31], was drastically higher (by 5-sixfold) in muscles from mice infected either with *P. berghei* (16.7 ± 7.7, *p *= 0.10133) or *P. chabaudi* (19.9 ± 11.9, *p *= 0.03887), when compared to the control group (3.6 ± 0.8) (Table 2).

## Discussion

Malaria is one of the most prevalent infectious disease in the world, causing splenomegaly and damage in other organs such as liver and kidney [8]. Remarkably, malaria infection can also cause muscle damage due to the combination of ischaemia, inflammation, and oxidative stress [8]. Considering the vital importance of the skeletal muscles as the largest organ-system in the body, comprising nearly 50% of its mass and largely responsible for the regulation and modulation of overall metabolism, the potential molecular mechanisms of *Plasmodium* infection affecting skeletal muscles of mice was first investigated. The expression of genes associate with inflammation, oxidative stress and atrophy were significantly altered in skeletal muscles infected with *Plasmodium* parasites. Supporting these results, higher concentrations of LMs directly associated to inflammatory responses were found in *Plasmodium* infected muscles, showing a crosstalk between LMs and gene expression.

Among all the genes analysed with the Mouse Signal Transduction Pathway Finder PCR array, the expression of six key genes, *Acsl*4, *Bmp2, Cebpd, Cdkn*1a, *Sqstm*1*, Stat*1 and *Txnrd*1, was significantly up-regulated in both *P. berghei* and *P. chabaudi* infected mice muscles, compared to the negative controls, and only one gene, *Acsl*4, was downregulated.

Significant changes of gene expression were observed in genes related to the inflammatory and immune response of the host. For instance, *Cebpd* gene encodes an important protein related to the regulation of genes involved in inflammatory and immune responses [32]. *Bmp*2 gene belongs to TGFβ signalling pathway, which has pleiotropic functions, including regulating immune response [33]. The gene *Txnrd*1 associated to oxidative stress pathways and is involved in the regulation of lipids metabolism via the PPAR signalling pathway [34].

*Stat1* gene it is a signal transducer and transcription activator that mediates cellular responses to interferons (IFNs), cytokines and other growth factors, being directly associated with the NF‐kB pathway [35]. *Sqstm*1 gene is associated to oxidative stress and to the NF-kB signalling pathway.

Another gene, *Cdkn1*, was upregulated in infected muscles with both *Plasmodium* species. This gene encoding a potent cyclin-dependent kinase inhibitor, is related to the p53 signalling pathway, and it might be an important intermediate by which p53/TP53 mediates its role as an inhibitor of cellular proliferation in response to DNA damage. The activation of NF‐kB and p53 can activate protein kinase (MAPK) pathways leading to skeletal muscle atrophy [36]. NF-kB is one of the most important signalling pathways linked to the loss of skeletal muscle mass in normal physiological and pathophysiological conditions [37]. The NF-κB pathway has long been considered a prototypical proinflammatory signalling pathway, largely based on the role of NF-κB in the expression of proinflammatory genes including cytokines, chemokines, and adhesion molecules, playing a key role in regulating the immune response to infection [38]. This result indicated that malaria infection may function through the NF‐kB pathway to regulate myoblast differentiation, since this pathway was affected by both *P. berghei* and *P. chabaudi* infections. These results corroborated with previous studies, which had shown a detrimental effects of malaria on skeletal muscles associated with significant decrease in content of key contractile proteins (reduced from 15 to 45%) in the skinned fibers [7], and also are in agreement with findings from other studies of the presence of muscle specific proteins in the circulation [13, 14, 16].

The results of gene array have shown a higher number of over regulated genes in skeletal muscles of *P. chabaudi* infected mice when compared to *P. berghei* infected mice. It is worth noting that the *Plasmodium* strains were selected according to their characteristics and effect on the experimental mice models. Infections with *P. berghei* ANKA strain can affect the brain and cause complications in laboratory mice, comparable to human cerebral malaria caused by *Plasmodium falciparum* [39], whereas malaria caused by *P. chabaudi*, strain CR, is not lethal, and defined as an experimental malaria self-control. This self-control characteristic of *P. chabaudi* may explain the higher number of over-expressed genes found in striated muscles infected with *P. chabaudi* compared to the low number of over expressed genes when the mice were infected with *P. berghei* ANKA strain.

It is known that the detrimental effects of malaria parasites on skeletal and cardiac muscles are related to their pathogenic mechanism. *Plasmodium berghei* is a mouse parasite, whose symptoms and complications are compared to the human parasite *P. falciparum*, that causes a severe malaria, provoked by microvascular sequestration of parasitized red blood cells causing microcirculatory obstruction [3], leading to decreasing oxygen delivery, obstruction of blood flow and tissue hypoxia [15]. Due to this aspect, skeletal muscle necrosis and rhabdomyolysis, which are directly or indirectly caused by muscle injury or death, have been reported in patients with severe falciparum malaria [3, 5, 6]. Considering that necropsies occur only in *P. falciparum* patients, but not in humans infected by the non-lethal *Plasmodium vivax* malaria, it was expected that some genes were only significantly altered in muscles of mice infected with the *P. chabaudi* parasites, which causes a not lethal infection comparable to *P. vivax*. For instance, the function related to *Mcl*1 gene, which encodes an anti-apoptotic protein, could explained the fact that in mice infected with this parasite do not go through an apoptosis process.

*Acsl4* gene that was downregulated in this study, encodes an isozyme of the long-chain fatty-acid-coenzyme synthase involved in the peroxisome proliferator-activated receptor (PPAR) signalling pathway and fatty acid pathway. PPARs play essential roles in the regulation of cellular differentiation, development, and metabolism of biomolecules such as carbohydrates, lipids and proteins of vertebrates [40–42]. This isozyme has marked preference for AA as its substrate, which is a polyunsaturated ω-6 fatty acid, related to a metabolic pathway directly related to inflammatory effects (Table 2).

The lipidomics profiling analysis revealed total 58 bioactive lipid mediators detectable in mice skeletal muscles, mostly related to AA and DHA metabolic pathways. These studies led us to conduct quantitative measurement of LMs in skeletal muscle models of malaria to elucidate the fundamental mechanisms of the different pathways of lipid metabolism in the skeletal muscle of mice infected with two types of malaria, a lethal mouse malaria caused by *P. berghei* and another form of mice malaria, non-lethal, caused by *P. chabaudi*. This may lead to better understanding of the cause and potential of treatments for harmful effects of malaria on skeletal muscle. Results of lipidomics quantification indicate significant differences in the levels of the determined LMs in the striated muscles of mice infected with both parasites, *P. berghei* and *P. chabaudi*, compared to the muscles of control mice (Table 2, Fig. 3). Few studies have shown evidence that lipid mediators may regulate skeletal muscle mass and function and potentially protect against muscle wasting in response to various infected diseases, but so far, no information about LM levels in *Plasmodium*-infected skeletal muscles has been reported. In the *P. berghei* infected mice group, the levels of 5 AA-metabolized LMs, including 6-keto-PGF_1α_, PGF_2α_, PGD_2_, PGA_2_, and12-HHT, were remarkably higher in muscles as compared to uninfected mice (Table 2, Fig. 3). On the other hand, EPA, DHA and AEA, derived from either ω-3 PUFA (EPA and DHA) or fatty acid ethanolamide, respectively, had statistically significant lower concentrations comparing to uninfected mice muscles. EPA concentration was statistically lower (*p* < 0.001) on muscles of mice infected with either malaria parasites. The same was found regarding to AEA concentration on muscles infected with either *Plasmodium* species (p < 0.001). Regarding to DHA, both infected malaria muscles were found on lower concentrations, but statistically lower (p < 0.05) only in muscles from *P. chabaudi* infected mice (Table 2, Fig. 3f–h).

Polyunsaturated fatty acids (PUFAs) are important constituents of the phospholipids of all cell membranes and generally considered to have beneficial effects. Some well-known LMs metabolized from PUFAs such as prostaglandins (PGs), thromboxanes (TXs) and leukotrienes (LTs) and related oxygenated derivatives have a wide range of biological actions including potent effects on inflammation [43]. However, it is believed that ω-3 and ω-6 PUFAs have opposing effects on metabolic functions in the body. Typically, ω-6 PUFAs are associated with inflammatory responses, constriction of blood vessels, and platelet aggregation [44]. In contrast, ω-3 PUFAs show anti-inflammatory and pro-resolving activities to resolve inflammation effectively, and alter the function of vascular and carcinogen biomarkers, thus reducing the risk of cancer [45–47]. Considering that malaria is known as an inflammatory disease, with recognized symptoms, such as fever and headache, signs, such as splenomegaly, and damage in other organs such as liver and kidney [8], the findings in this study corroborated with those assessments, since all LMs found on significantly higher concentrations compared to the controls are from ω-6 pathway (AA and LA pathways, Table 2), while the lipids found at remarkably lower concentrations comparing to the controls are related to ω-3 pathway (EPA and DHA pathways, Table 2). Accordingly, a significantly higher AA/EPA ratio was also found in *Plasmodium* infected mice compared to uninfected ones. This significantly increased AA/EPA ratios may also suggest an association between malaria infection and skeletal muscle depletion.

The results of lipid profiles also indicate the potentially increased peroxidation in skeletal muscles infected with malaria parasites. Recently, hydroxyoctadecadienoic acids (HODEs) such as 13-HODE and 9-HODE have been reported as lipid peroxidation biomarkers [29]. HODEs are stable oxidation products of linoleic acid (LA, 18:2, ω-6), a most abundant PUFA in vivo, by 15-lipoxygenase-1 (15-LOX-1) followed by glutathione peroxidases and phospholipases [28, 48]. HODEs accumulate in atherosclerotic lesions and exert protective effects in atherogenesis by modulating macrophage lipid accumulation and inflammatory mediator generation through peroxisome proliferator-activated receptor-γ (PPAR-γ) activation [49, 50]. Elevated levels of HODEs is always linked to oxidative stress-related diseases such as diabetes [49]. In the present study, both 13-HODE and 9-HODE showed increased levels in skeletal muscles from malaria infected mouse, confirming previous data showing that malaria infection induces a combination of inflammation and oxidative damage in skeletal muscles [14, 15]. These data are also corroborated by the gene array results, which showed an upregulated expression of *Sqstm*1 gene, associated to oxidative stress pathway.

Therefore, profiles of targeted lipid mediators obtained in this study provided crucial information related to the aspects of skeletal muscle infected with the malaria parasite *P. berghei* and *P. chabaudi*, with insights on the quantification of LMs involved with essential physiological functions ranging from tissue repair and inflammation due to malaria infection. The study further points to a crosstalk between AA-related lipid mediators and *Acsl*4 gene as a potential cellular mechanism of sustained inflammation in muscles of *Plasmodium* infected mice.

## Conclusion

**Fig. 4:**
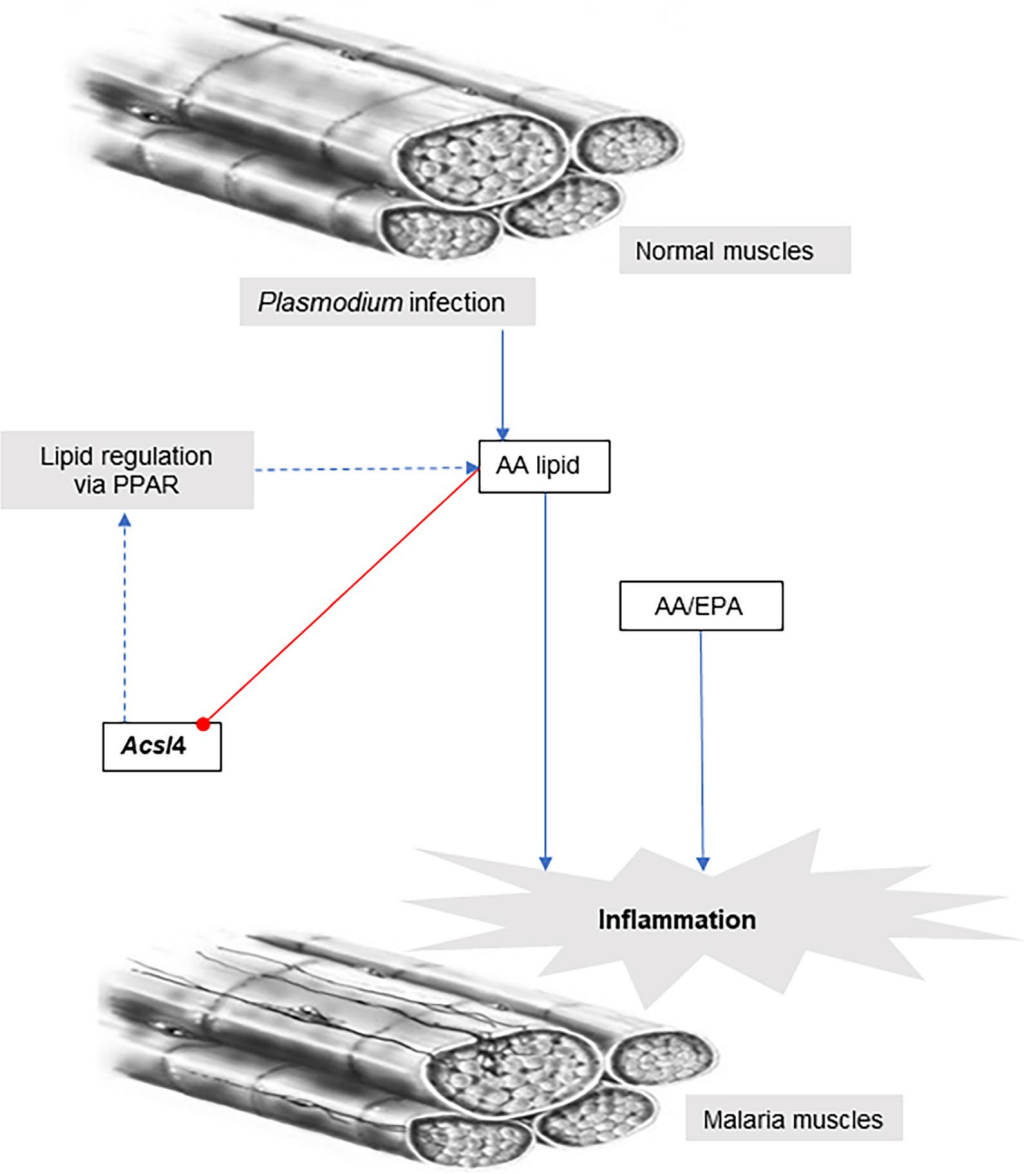
Schematic representation of arachnoid acid (AA) related lipids downregulating *Acsl*4 gene and modulating inflammatory process in malaria infected skeletal muscles. Blue arrow-ended line: stimulation/activation; red circle-ended line: inhibition; dash line: PPAR signalling pathway

The gene profiling analyses showed expression of key genes altered by malaria infection associate with inflammation, oxidative stress, and atrophy. In support to these results, the lipidomics analyses revealed higher concentrations of LMs directly related to inflammatory responses, while on the levels of LMs crucial in resolving inflammation and tissue repair reduced significantly. Figure 4 shows a proposed model for the potential mechanism between AA derived lipid mediators and the *Acsl*4 gene; a gene directly related to inflammation process of malaria-infected muscles. The high concentration of the AA related lipid mediators found in malaria infected mice may act downregulating the *Acsl*4 [51], promoting the inflammatory response. Therefore, the present study helped to increase the knowledge of mechanisms underlying molecular mechanisms of malaria-induced muscle damage, targeting key molecules, such as genes and lipids, associated and leading to muscle damage. Together, this information could be useful in future works to provide better monitoring of disease progression and development of specific interventions for the mitigation of long-term chronic effects on skeletal muscle function.

## Supplementary information

**Additional file 1: Table S1.** Lipid profiling (% to the control) in striated muscles from mice with or without different malaria infections and the associated metabolic pathways.

## Data Availability

The datasets used and/or analysed during the current study are available from the corresponding author on reasonable request.

## References

[1] WHO (2017). World malaria report 2017.

[2] Orem JN, Kirigia JM, Azairwe R, Kasirye I, Walker O (2012). Impact of malaria morbidity on gross domestic product in Uganda. Int Arch Med..

[3] Da Silva HJ, Goonetilleke AK, Senaratna N, Ramesh N, Jayawickrama US, Jayasinghe KS (1988). Skeletal muscle necrosis in severe falciparum malaria. BMJ.

[4] Miller KD, White NJ, Lott JA, Roberts JM, Greenwood BM (1989). Biochemical evidence of muscle injury in African children with severe malaria. J Infect Dis.

[5] Taylor WR, Prosser DI (1992). Acute renal failure, acute rhabdomyolysis and falciparum malaria. Trans R Soc Trop Med Hyg.

[6] Knochel JP, Moore GE (1993). Rhabdomyolysis in malaria. N Engl J Med.

[7] Brotto MA, Marrelli MT, Brotto LS, Jacobs-Lorena M, Nosek TM (2005). Functional and biochemical modifications in skeletal muscles from malarial mice. Exp Physiol.

[8] Marrelli MT, Brotto M (2016). The effect of malaria and anti-malarial drugs on skeletal and cardiac muscles. Malar J..

[9] Takaya S, Kutsuna S, Suzuki T, Komaki-Yasuda K, Kano S, Ohmagari N (2018). Case Report: *Plasmodium knowlesi* Infection with Rhabdomyolysis in a Japanese traveler to Palawan, the Philippines. Am J Trop Med Hyg.

[10] Herr J, Mehrfar P, Schmiedel S, Wichmann D, Brattig NW, Burchard GD (2011). Reduced cardiac output in imported *Plasmodium falciparum* malaria. Malar J..

[11] Janka JJ, Koita OA, Traoré B, Traoré JM, Mzayek F, Sachdev V (2010). Increased pulmonary pressures and myocardial wall stress in children with severe malaria. J Infect Dis.

[12] Costenaro P, Benedetti P, Facchin C, Mengoli C, Pellizzer G (2011). Fatal myocarditis in mouse of *Plasmodium falciparum* infection: case report and review of cardiac complications in malaria. Case Rep Med..

[13] Ehrhardt S, Mockenhaupt FP, Anemana SD, Otchwemah RN, Wichmann D, Cramer JP (2005). High levels of circulating cardiac proteins indicate cardiac impairment in African children with severe *Plasmodium falciparum* malaria. Microbe Infect..

[14] Ehrhardt S, Wichmann D, Hemmer CJ, Burchard GD, Brattig NW (2004). Circulating concentrations of cardiac proteins in complicated and uncomplicated *Plasmodium falciparum* malaria. Trop Med Int Health..

[15] Yeo TW, Lampah DA, Kenangalem E, Tjitra E, Price RN, Anstey NM (2013). Impaired skeletal muscle microvascular function and increased skeletal muscle oxygen consumption in severe falciparum malaria. J Infect Dis.

[16] Fern EB, McNurlan MA, Garlick PJ (1985). Effect of malaria on rate of protein synthesis in individual tissues of rats. Am J Physiol.

[17] Liu M, Saeki K, Matsunobu T, Okuno T, Koga T, Sugimoto Y (2014). 12-Hydroxyheptadecatrienoic acid promotes epidermal wound healing by accelerating keratinocyte migration via the BLT2 receptor. J Exp Med.

[18] Murakami M (2011). Lipid mediators in life science. Exp Anim.

[19] Hellmann J, Tang Y, Spite M (2012). Proresolving lipid mediators and diabetic wound healing. Curr Opin Endocrinol Diabetes Obes.

[20] Altmann R, Hausmann M, Spottl T, Gruber M, Bull AW, Menzel K (2007). 13-Oxo-ODE is an endogenous ligand for PPARgamma in human colonic epithelial cells. Biochem Pharmacol.

[21] Ferre P (2004). The biology of peroxisome proliferator-activated receptors: relationship with lipid metabolism and insulin sensitivity. Diabetes.

[22] Kliewer SA, Sundseth SS, Jones SA, Brown PJ, Wisely GB, Koble CS (1997). Fatty acids and eicosanoids regulate gene expression through direct interactions with peroxisome proliferator-activated receptors alpha and gamma. Proc Natl Acad Sci USA.

[23] Mo C, Romero-Suarez S, Bonewald L, Johnson M, Brotto M (2012). Prostaglandin E2: from clinical applications to its potential role in bone- muscle crosstalk and myogenic differentiation. Recent Pat Biotechnol.

[24] Markworth JF, Maddipati KR, Cameron-Smith D (2016). Emerging roles of pro-resolving lipid mediators in immunological and adaptive responses to exercise-induced muscle injury. Exerc Immunol Rev..

[25] Hoffmann EJ, Weidanz WP, Long CA (1984). Susceptibility of CXB recombinant inbred mice to murine plasmodia. Infect Immun.

[26] Huang J, Hsu YH, Mo C, Abreu E, Kiel DP, Bonewald LF (2014). METTL21C is a potential pleiotropic gene for osteoporosis and sarcopenia acting through the modulation of the NF-κB signaling pathway. J Bone Miner Res.

[27] Wang Z, Bian L, Mo C, Kukula M, Schug KA, Brotto M (2017). Targeted quantification of lipid mediators in skeletal muscles using restricted access media-based trap-and-elute liquid chromatography-mass spectrometry. Anal Chim Acta.

[28] Yoshida Y, Niki E (2006). Bio-markers of lipid peroxidation in vivo: hydroxyoctadecadienoic acid and hydroxycholesterol. BioFactors.

[29] Wallert M, Ziegler M, Wang X, Maluenda A, Xu X, Yap ML (2019). α-Tocopherol preserves cardiac function by reducing oxidative stress and inflammation in ischemia/reperfusion injury. Redox Biol..

[30] Kitagawa M, Haji S, Amagai T (2017). Elevated Serum AA/EPA Ratio as a predictor of skeletal muscle depletion in cachexic patients with advanced gastro-intestinal cancers. Vivo..

[31] Tutino V, De Nunzio V, Caruso MG, Veronese N, Lorusso D, Di Masi M (2019). Elevated AA/EPA ratio represents an inflammatory biomarker in tumor tissue of metastatic colorectal cancer patients. Int J Mol Sci..

[32] Moore F, Santin I, Nogueira TC, Gurzov EN, Marselli L, Marchetti P (2012). The transcription factor C/EBP delta has anti-apoptotic and anti-inflammatory roles in pancreatic beta cells. PLoS One.

[33] Chen D, Zhao M, Mundy GR. Bone morphogenetic proteins. Growth Factors. 2004;22:233-41. Review. 10.1080/08977190412331279890 15621726

[34] Peng X, Giménez-Cassina A, Petrus P, Conrad M, Rydén M, Arnér ESJ (2016). Thioredoxin reductase 1 suppresses adipocyte differentiation and insulin responsiveness. Sci Rep..

[35] Chen K, Liu J, Liu S, Xia M, Zhang X, Han D (2017). Methyltransferase SETD2-mediated methylation of STAT1 is critical for interferon antiviral activity. Cell.

[36] Guttridge DC (2004). Signaling pathways weigh in on decisions to make or break skeletal muscle. Curr Opin Clin Nutr Metab Care..

[37] Li H, Malhotra S, Kumar A (2008). Nuclear factor-kappa B signaling in skeletal muscle atrophy. J Mol Med.

[38] Lawrence T (2009). The nuclear factor NF-kappaB pathway in inflammation. Cold Spring Harb Perspect Biol..

[39] Franke-Fayard B, Fonager J, Braks A, Khan SM, Janse CJ (2010). Sequestration and tissue accumulation of human malaria parasites: can we learn anything from rodent models of malaria?. PLoS Pathog.

[40] Berger J, Moller DE (2002). The mechanisms of action of PPARs. Annu Rev Med.

[41] Belfiore A, Genua M, Malaguarnera R (2009). PPAR-gamma agonists and their effects on IGF-I receptor signaling: implications for cancer. PPAR Res..

[42] Feige JN, Gelman L, Michalik L, Desvergne B, Wahli W (2006). From molecular action to physiological outputs: peroxisome proliferator-activated receptors are nuclear receptors at the crossroads of key cellular functions. Prog Lipid Res.

[43] Yang R, Chiang N, Oh SF, Serhan CN. Metabolomics-lipidomics of eicosanoids and docosanoids generated by phagocytes. Curr Protoc Immunol. 2011;Chapter 14:Unit 14.26. 10.1002/0471142735.im1426s95 PMC3321370 22048801

[44] Calder PC (2013). Omega-3 polyunsaturated fatty acids and inflammatory processes: nutrition or pharmacology?. Br J Clin Pharmacol.

[45] Ricciotti E, FitzGerald GA (2011). Prostaglandins and inflammation. Arterioscler Thromb Vasc Biol.

[46] Cottin SC, Sanders TA, Hall WL (2011). The differential effects of EPA and DHA on cardiovascular risk factors. Proc Nutr Soc..

[47] Yui K, Imataka G, Nakamura H, Ohara N, Naito Y (2015). Eicosanoids Derived from arachidonic acid and their family prostaglandins and cyclooxygenase in psychiatric disorders. Curr Neuropharmacol.

[48] Zuo X, Wu Y, Morris JS, Stimmel JB, Leesnitzer LM, Fischer SM (2006). Oxidative metabolism of linoleic acid modulates PPAR-beta/delta suppression of PPAR-gamma activity. Oncogene.

[49] Vangaveti V, Baune BT, Kennedy RL (2010). Hydroxyoctadecadienoic acids: novel regulators of macrophage differentiation and atherogenesis. Ther Adv Endocrinol Metab..

[50] Itoh T, Fairall L, Amin K, Inaba Y, Szanto A, Balint BL (2008). Structural basis for the activation of PPARgamma by oxidized fatty acids. Nat Struct Mol Biol.

[51] Kan CF, Singh AB, Stafforini DM, Azhar S, Liu J (2014). Arachidonic acid downregulates acyl-CoA synthetase 4 expression by promoting its ubiquitination and proteasomal degradation. J Lipid Res.

