# Supplementary material for: The skeletal muscles of mice infected with Plasmodium berghei and Plasmodium chabaudi reveal a crosstalk between lipid mediators and gene expression
Source: Malar J. 2020 Jul 14;19:254. doi: 10.1186/s12936-020-03332-3 (PMC7362477; doi:10.1186/s12936-020-03332-3)
Supplement: Supplementary file 1 — Additional file 1: Table S1. Lipid profiling (% to the control) in striated muscles from mice with or without different malaria infections and the associated metabolic pathways. [file 12936_2020_3332_MOESM1_ESM.docx]

**Table S1.** Lipid profiling (% to the control) in striated muscles from mice with or without different malaria infections and the associated metabolic pathways.

| Metabolic pathways | Lipid Mediators | Control | *P. chabaudi* | *P. berghei* |
| --- | --- | --- | --- | --- |
| Arachidonic acid (AA) | 8-iso-13,14-dihydro-15-keto-PGF2a | 100±29.0 | 547.5±235.5** | 308.8±83.8 |
|  | 5S,6R-LXA4 | 100±23.1 | 538.7±257.9** | 359.6±101.0 |
|  | 5S,6S-LXA4 | 100±13.1 | 808.6±429.9** | 489.4±153.8* |
|  | 5,15-DiHETE | 100±25.8 | 515.7±270.4* | 381.9±152.0 |
|  | 11,12-EET-EA | 100±107.7 | 1063.0±729.0* | 288.9±196.3 |
|  | 15-HETE | 100±16.9 | 348.0±163.5* | 261.6±46.9 |
|  | 11-HETE | 100±18.2 | 328.6±183.2* | 230.1±36.6 |
|  | 5-HETE | 100±22.6 | 268.0±121.9* | 206.0±36.2 |
| Eicosadienoic acid (EDA) | 15-HEDE | 100±13.2 | 401.2±398.0* | 209.1±66.5 |
| Docosahexaenoic acid (DHA) | 10,17-DiHDoHE | 100±29.0 | 332.4±139.8** | 258.2±68.8 |
|  | 10-HDoHE | 100±45.0 | 231.1±90.1* | 209.7±50.1 |
|  | 13-HDoHE | 100±40.2 | 256.1±93.0* | 241.6±60.9* |
|  | 14-HDoHE | 100±37.7 | 227.2±78.3* | 226.1±45.2* |
|  | 16-HDoHE | 100±33.5 | 196.9±73.6 | 213.5±45.8* |
|  | 17-HDoHE | 100±33.1 | 188.8±70.2 | 209.2±44.4* |
|  | 20-HDoHE | 100±37.1 | 226.5±83.0* | 232.0±61.4* |

Mean ± SD, n=5. One-way ANOVA with Tukey post-hoc test (α = 0.05) was applied to compare means between multiple groups. *p≤0.05, and **p≤0.01 represent statistically significant difference between uninfected mice (Control) and *P. berghei* or *P. chabaudi* infected mice.
